# Influence of the Long-Range Transport of Siberian Biomass Burnings on Air Quality in Northeast China in June 2017

**DOI:** 10.3390/s23020682

**Published:** 2023-01-06

**Authors:** Li Sun, Lei Yang, Dongdong Wang, Tiening Zhang

**Affiliations:** 1 Institute of Atmospheric Environment, China Meteorological Administration, Shenyang 110166, China; 2 Liaoning Weather Modification Office, Shenyang 110166, China; 3 Liaoning Meteorological Disaster Monitoring and Early Warning Centre, Shenyang 110166, China

**Keywords:** biomass burning, long-range transport, air quality, satellite, remote sensing

## Abstract

Biomass burning (BB) emits a large volume of trace gases and aerosols into the atmosphere, which can significantly affect the earth’s radiative balance and climate and has negative impacts on air quality and even human health. In late June 2017, an intense BB case, dominated by forest and savanna fires, occurred in Siberia, and it affected the air quality of Northeast China through long-range transport. Here, multisatellite remote-sensing products and ground-based PM_2.5_ measurements are used to evaluate the influence of the Siberian smoky plume on Northeast China. The results show that the BB was intense at the early stage when the daily fire count and average fire radiative power exceeded 300 and 200 MW, respectively. The maximum daily fire count reached 1350 in Siberia, and the peak value of instantaneous fire radiative power was as high as 3091.5 MW. High concentrations of CO and aerosols were emitted into the atmosphere by the BB in Siberia. The maximum daily mean values of the CO column concentration and aerosol optical depth (AOD) increased by 3 × 10^17^ molec·cm^2^ and 0.5 compared with that during the initial BB stage. In addition, the BB released a large number of absorptive aerosols into the atmosphere, and the UV aerosol index (UVAI) increased by five times at the peak of the event in Siberia. Under the appropriate synoptic conditions and, combined with pyroconvection, the smoky plume was lifted into the upper air and transported to Northeast China, affecting the air quality of Northeast China. The daily mean values of CO concentration, AOD, and UVAI in Northeast China increased by 6 × 10^17^ molec·cm^2^, 0.5, and 1.4, respectively, after being affected. Moreover, the concentration of the surface PM_2.5_ in Northeast China approximately doubled after being affected by the plume. The results of this study indicate that the air quality of Northeast China can be significantly affected by Siberian BBs under favorable conditions.

## 1. Introduction

Biomass burning (BB) associated with human land-use activities, as well as naturally occurring wildfires, plays an important role in the environment and climate change by emitting large amounts of smoke aerosols and trace gases into the atmosphere [1,2,3,4]. As one of the types of BBs, forest fires contribute to 4–12% of the biomass annually burned worldwide [5]. These occur over 200,000 times per year, with burned areas of 3.5 to 4.5 million km^2^ around the world, which is greater than half of the land areas of Australia [6]. 

Boreal forests comprise 30% of the world’s forested area, and Russia alone contains more than two-thirds of the boreal forests, with the majority of the rest in Canada and Alaska [7,8]. The annual area burned in Russian forests is many times that of North America [9]. Moreover, the boreal fire frequency and size are expected to increase as a result of climate warming in high northern latitudes [10]. The trace gases and aerosols generated from large-scale boreal zone burning can contribute to regional and hemisphere air pollution [11,12,13,14,15]. In the past few decades, the impacts of long-range transported Siberian plumes on air quality in Mongolia, Korea, Japan, and the Pacific Northwest have been intensely investigated [16,17,18,19,20,21]. Due to the long-range transport of Siberian BB emissions in 2003, high aerosol optical depth (AOD) in the range of 2.0–4.0 and an increased surface PM_10_ concentration of up to 258 μg·m^−3^ were observed in Korea [19]. The fires even resulted in the enhancement of summer background CO and O_3_ of 23–37 and 5–9 ppbv, respectively, at 10 sites in Alaska, Canada, and the Pacific Northwest [4]. The long-range transport of a Siberian smoke plume in July 2012 affected air quality in the Pacific Northwest, enhancing O_3_ by 34–44 ppbv and PM_2.5_ by 10–32 μg·m^−3^ in the Interior of British Columbia and at the Whistler Peak High Elevation Site [20]. 

Because of its close proximity to the location of Siberia, Northeast China must be affected by the plumes from Siberian BBs. However, due to limited research, little is known about the impact of the long-range transport of BB emissions from Siberia on the air quality and aerosol optical properties in Northeast China [17,22]. In June 2017, the long-range transport of smoke aerosols from Siberian BBs to Northeast China was observed. In this study, the fire activity and its impacts on the environment were studied using satellites and surface observations. The regional enhancements of CO, aerosol concentration, and variation in aerosol optical properties were focused on. This paper is organized as follows. In Section 2, the study area and data are described. The results are presented in Section 3. Discussions are presented in Section 4. Conclusions follow in Section 5.

## 2. Study Area and Data 

### 2.1. Study Area

**Figure 1:**
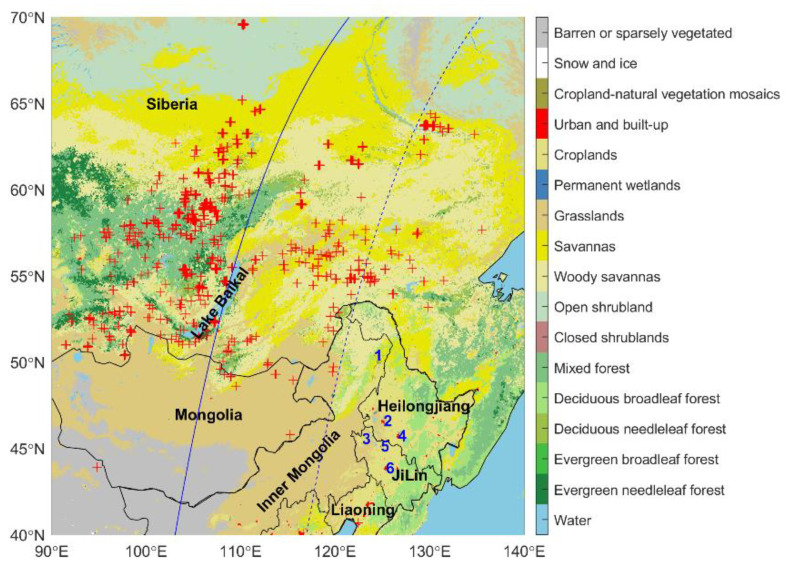
The MODIS land cover map and active fire points (plus simple: red color) measured by Aqua MODIS from 20 June to 30 June 2017 in Northeast Asia. The spatial distribution of six PM2.5 stations is marked with blue numbers. The solid blue line is the ground track of the CALIPSO satellite on 25 June 2017, and the dashed blue line is the track on 26 June 2017.

In this study, the atmospheric environment in Northeast Asia was focused on. The specific area is shown in Figure 1. Siberia is a vast area in Russia. During the BB event in June 2017, the fires were mainly active in Central and Eastern Siberia, where the forest is distributed north and west of Lake Baikal and the rest of the area is dominated by savannas. Northeast China includes the Heilongjiang, Jilin, Liaoning provinces, and the eastern part of the Inner Mongolia Autonomous Region. In this study, only the area east of 118° N in China was referred to as Northeast China. Northeast China borders Siberia from Russia in the north and Mongolia in the west. 

### 2.2. MODIS Fire, Aerosol, and Land Cover Products

The launch of NASA’s Terra in late 1999 and Aqua in the middle of 2002 marked a significant step forward in the ability to monitor fire activity and atmospheric pollutants from space at a global scale [23]. The Moderate Resolution Imaging Spectroradiometer (MODIS) onboard Terra and Aqua was designed for the observation of actively burning fires and aerosol loading twice per day under relatively cloud-free conditions [24]. The global distribution of fire counts and fire radiative power (FRP) can be inferred from MODIS observations. The Collection 6 daily level 3 fire product MYD14A1(Aqua) with a spatial resolution of 1 km was used to show the distribution of active fires in June 2017 in northeast Asia. Only the fire pixels with high confidence were extracted.

Aqua MODIS Collection 6 level 2 aerosol optical depth product at 550 nm (AOD) was used to reveal the variation in aerosol loading during the BB episode in June 2017. We use the merged AOD product retrieved by the combined dark target and deep blue algorithm since it provides a more gap-filled data set than the individual algorithms alone. 

The Collection 6 level 3 yearly Terra and Aqua MODIS combined land cover product with a spatial resolution of 0.05° (MCD12C1) was used to show the land cover types for the burning area. It contains six classification schemes, and the primary land cover scheme, International Geosphere Biosphere Program (IGBP), was selected [25,26]. 

### 2.3. Meteorological Data

Meteorological conditions play an important role in plume transportation and air quality in downwind [17]. In this study, the Final Analysis (FNL) dataset provided by the National Centers for Environmental Prediction (NCEP) was used to explore the variation in synoptic conditions. FNL is a global gridded dataset with a 1° spatial resolution and 26 vertical levels from the sea surface to 10 hPa every six hours. 

### 2.4. AIRS CO Concentration

Atmospheric Infrared Sounder (AIRS) was launched on 4 May 2002 on board Aqua with a cross-track scanning swath width of 1650 km. The Version 6 level 3 total column CO concentration measured by AIRS was used as a tracer to the BB. AIRS can retrieve CO concentration for cloud fraction by as high as 80% [27].

### 2.5. CALIPSO Aerosol Vertical Profile

The Cloud-Aerosol Lidar with Orthogonal Polarization on board the CALIPSO platform provides information on the vertical distribution of aerosols, as well as their optical properties over the globe with a high spatial resolution (since June 2006) [28]. The Vertical Feature Mask (VFM), one of CALIPSO level 2 products, classifies contiguous atmospheric regions of backscatter as different layers, such as dust, smoke, cloud, and clear air [29]. To investigate aerosol transport and identify aerosol types over Northeast Asia, the VFM product of version 4.20 data was used.

### 2.6. OMI Aerosol Index

The measurement of Ozone Monitoring Instrument (OMI) on the Aura satellite can be used to derive the concentration of trace gases, as well as aerosol properties and clouds [30,31]. The level 3 near-UV aerosol index (UVAI) with a spatial resolution of 1° × 1°collected by OMI onboard the Aura satellite was used to analyze the aerosol variation. Since early 2008, the sensor’s viewing capability has been affected by a loss of spatial coverage, which is referred to as “row anomaly”. An improved version of the OMAERUV algorithm has been incorporated into the products [30]. UVAI is strongly correlated with atmospheric aerosol load from UV-absorbing aerosols, such as carbonaceous aerosols, mineral dust, and volcanic ash [32]. It has been widely used to explore the spatiotemporal variation of BB and dust aerosols.

### 2.7. PM_2.5_ Data

Daily PM_2.5_ (particle with an aerodynamic diameter less than 2.5 μm) data are available from the China Air Quality Monitoring and Analysis Platform (https://www.aqistudy.cn/historydata/,accessed on 15 September 2022). To explore the influence of long-range transported plume on air quality in Northeast China, the daily PM_2.5_ concentration at six sites in Northeast China was used. The locations of the sites are shown in Figure 1. 

## 3. Results

### 3.1. Overview of Siberian 2017 BB Event

**Figure 2:**
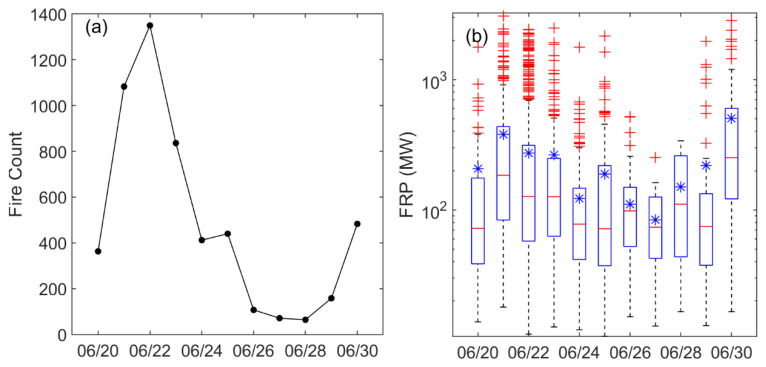
Daily fire counts (**a**) and fire radiative power (FRP) (**b**) in Siberia, monitored by Aqua MODIS from 20 June to 30 June 2017. The central bar in (**b**) is the median and the lower and upper limits are the first and third quartiles, respectively. The lines extending vertically from the box indicate the distribution spread, with the length being 1.5 times the difference between the first and third quartiles. Observations falling beyond the limits of those lines are indicated by plus symbols. The asterisk symbols represent geometric means.

A wide-ranging BB event in Siberia was monitored by MODIS during 20–30 June 2017 (Figure 1). As shown in Figure 1, active fires occurred in the forests and savannas. The fire spots were mainly scattered in Central Siberia and Eastern Siberia, covering nearly 40 longitudes. Figure 2 shows the daily fire counts and FRP in Siberia. The peak of daily fire counts occurred on 22 June 2017, with 1350, and the highest instantaneous FRP occurred on 21 June and was as high as 3091.4 MW, more than five times the multiannual average [18]. Before 24 June, the daily fire count and average FRP exceeded 300 and 200 MW, respectively, indicating high-intensity burning. The high FRP indicated a large fuel load in forests and savannas [33]. In addition, there were very few fire spots in Northeast China, and the maximum number of daily fires during the study period was five, mainly concentrated on the border between China and Mongolia. 

**Figure 3:**
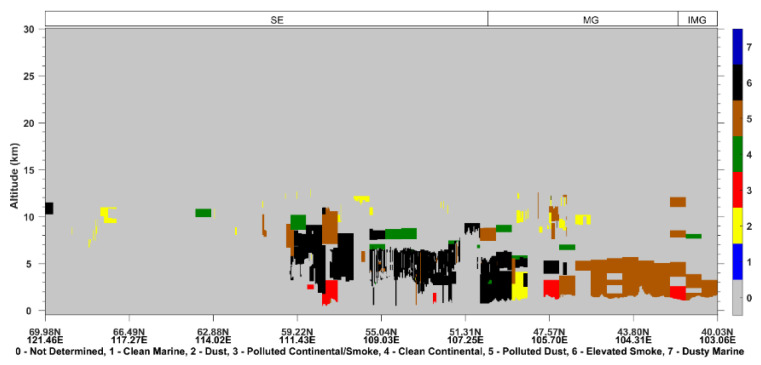
25 June 2017: CALIOP-derived vertical profile of the vertical feature mask of aerosol over the ground track (solid blue line) shown in Figure 1. CALIOP successively overpassed Siberia (SE), Mongolia (MG), and Inner Mongolia Autonomous Region of China (IMG).

The CALIOP satellite passed over the burning area of Siberia, especially Lake Baikal, in the early morning of 25 June 2017 (Figure 1). As shown in Figure 2, the total daily fire counts and mean FRP were 440 and 188.4 MW on 25 June. The aerosol derived from CALIOP showed that the elevated smoke dominated the atmosphere between 1–10 km over Lake Baikal (Figure 3). The deep aerosol layer might be attributed to strong pyroconvective lofting due to the high thermal convective energy released from burning [14,34]. The BB aerosol lofted to the free troposphere is less likely to be removed and can have a longer atmospheric lifetime [35].

### 3.2. Long-Range Transportation Trajectory of Siberian Plume 

**Figure 4:**
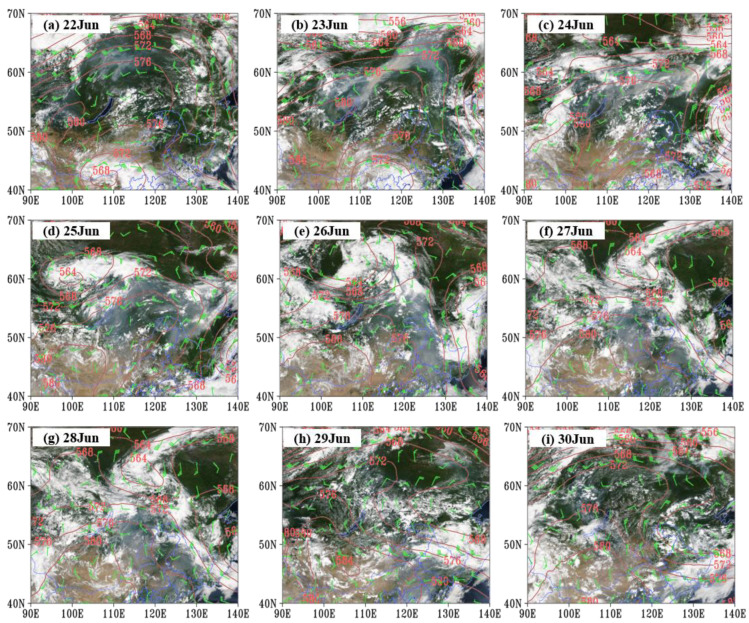
The geopotential height at 500 hPa and wind field at 700 hPa and Aqua MODIS daytime true color images at 06:00 GMT from 22 June to 30 June 2017 in Northeast Asia.

The MODIS true color imagery and the weather pattern in Northeast Asia during 22–30 June 2017 are shown in Figure 4. A shear line dominated the west side of Lake Baikal at 700 hPa on 22 June, resulting in the upward movement of the atmosphere, and it moved to the north side of Lake Baikal on 23 June. In addition, during 22–24 June, there was a high-pressure ridge from southwest to northeast at 500 hPa over the combustion area in Siberia. The combustion area was affected by the westerly wind at the top of the high-pressure ridge. The dynamic uplifting, coupled with pyroconvection, carried the smoke to a high altitude and transported it eastward. On 24 June, the plume was transported to the top of the high-pressure ridge at 572 dagpm (near 130° E, 60° N). During 24–25 June, a cold vortex system appeared in Northern Siberia and gradually moved eastward (Figure 4c,d). In addition, under the blocking effect of the Okhotsk Sea cold vortex, the contour gradient and meridional wind in the front of the high ridge over Siberia increased significantly. Consequently, the smoke diffused to Northeast China under the enhanced northerly airflow in front of the high-pressure ridge. The MODIS true color imagery showed a clearly southward transported smoke plume from 24 to 25 June 2017, and it arrived in northeastern China on 25 June. Although the cold vortex system at 60° N moved eastward, and the blocking situation of the cold vortex over the Okhotsk Sea disappeared on 28 June, the northwest-southeast high ridge over Siberia was established again on 29 June. Influenced by the northerly airflow, the smoke plume was transported southward and affected northern and central parts of Northeast China. Furthermore, it should be noted that during the transmission of the smoke, Northeast China was in front of the high-pressure ridge, which resulted in large-scale subsidence of air. Therefore, the Siberian smoky air was transported to the surface and had a significant influence on regional air quality. 

### 3.3. Impact of Siberian Plume on Trace Gases and Aerosols 

**Figure 5:**
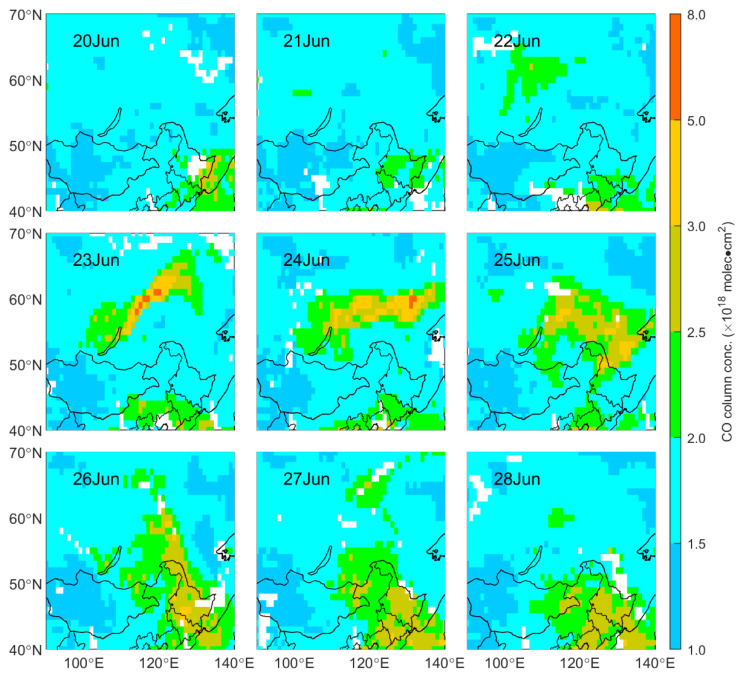
Spatial distribution of CO total column concentration (unit: molec·cm^2^) from 20 June to 28 June 2017 in Northeast Asia.

**Figure 6:**
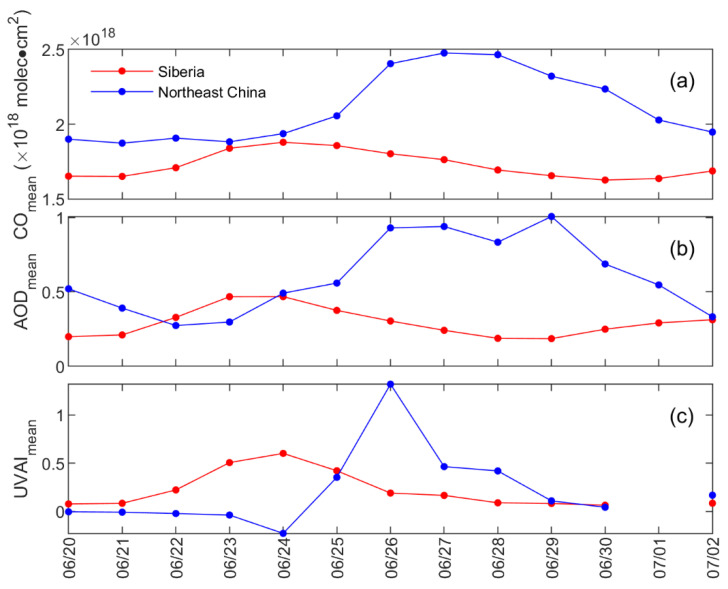
Daily mean values of (**a**) CO column concentration, (**b**) AOD, and (**c**) UVAI in Siberia and Northeast China.

BB can release a large volume of gaseous pollutants, especially carbon oxides dominated by CO [36,37]. Figure 5 shows the spatial distribution of CO column concentration during this episode. The daily mean value of CO concentration in Siberia is shown in Figure 6a. The high-value area of CO column concentration was consistent with the evolution of the smoke plume displayed by MODIS (Figure 5). Before 22 June, the daily mean value of the CO concentration was low in Siberia: ~1.6 × 10^18^ molec·cm^2^. From 22 June to 23 June, the CO column concentrations increased due to the intense fire activity in Siberia. The concentrations of CO were all below 2.0 × 10^18^ molec·cm^2^ in the unaffected areas of Siberia, while high values were recorded in the combustion area, with the maximum appearing on 23 June reaching 7.4 × 10^18^ molec·cm^2^. On 24 June, during the eastward transmission of the BB plume, the area with a high CO concentration widened and was located on the east side of the plume. The peak concentration of CO dropped to 5.7 × 10^18^ molec·cm^2^ on 24 June, but the daily mean concentration of CO reached a maximum of 1.9 × 10^18^ molec·cm^2^ in Siberia. Compared with the daily mean concentration of CO before 22 June, it increased by 3.0 × 10^17^ molec·cm^2^ (~19%) on 24 June. 

For the CO in Northeast China, its concentration was low before 25 June, mostly below 2.5 × 10^18^ molec·cm^2^, especially in Heilongjiang and the northeastern Inner Mongolia Autonomous Region of China. The daily mean was approximately 1.9 × 10^18^ molec·cm^2^ before 25 June (Figure 6a). On 25 June, the polluted plume was transmitted to Northeast China, and the CO column concentration in the north of Northeast China began to rise rapidly, with the maximum CO concentration increasing to 3.2 × 10^18^ molec·cm^2^. After that, it gradually spread to the central and southern regions of Northeast China, and the area with a high CO concentration continued to expand; the daily mean CO concentration reached a maximum at 2.5 × 10^18^molec·cm^2^ on 27 June, increasing by 6 × 10^17^molec·cm^2^ (~32%) compared with that before 25 June.

**Figure 7:**
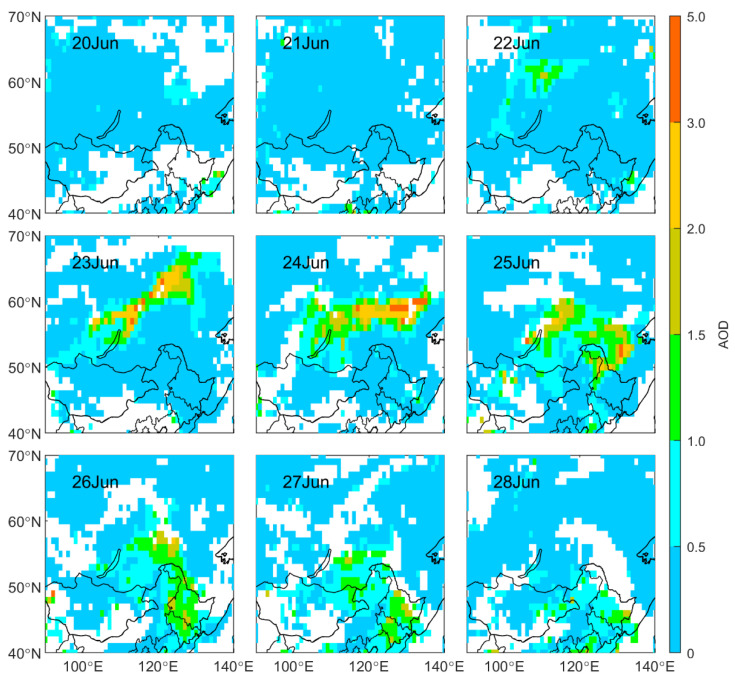
Spatial distribution of AOD monitored by Aqua MODIS from 20 June to 28 June 2017 in Northeast Asia.

BB not only releases a large number of gases but also produces abundant aerosols [17,18]. As shown in Figure 7, the AOD in the unaffected areas in Siberia was mostly lower than 0.5, with a daily mean AOD remaining at 0.2 before 22 June (Figure 6b). From 22 June to 23 June, the peak value of AOD in Siberia increased from 2.0 to 4.2, indicating a massive accumulation of aerosols. During the eastward transmission, the peak concentration of AOD reached 4.7 on 24 June. The daily mean AOD in Siberia peaked on 24 June, 2.5 times that before June 22. For AOD in Northeast China, it was around 0.4 before the smoky plume’s arrival, and it reached a maximum of 2.2 on 25 June after being affected by the plume. The daily mean AOD remained at a high level during 26–29 June (Figure 6b). There were two peaks during this period; one occurred on 27 June and another on 29 June. The AOD peak on 27 June was attributed to the impact of the Siberian plume, and the other was due to the long-range transport of the plume from Siberia and an airmass from the North China Plain (Figure 4h). The daily mean AOD more than doubled compared with that before the Siberian plume arrived.

**Figure 8:**
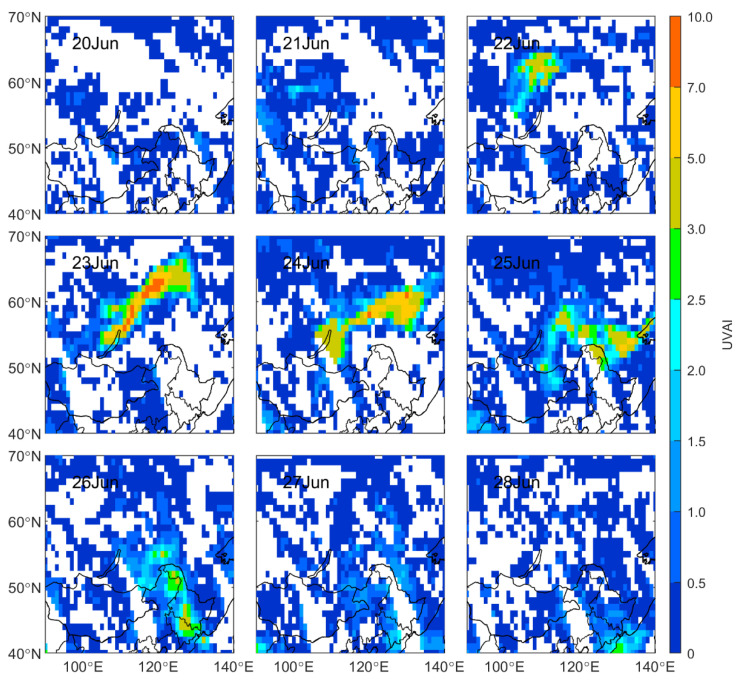
Spatial distribution of OMI UVAI from 20 June to 28 June 2017 in Northeast Asia.

In order to explore the impact of the BB plume on aerosol optical properties, the UVAI measured by OMI during the episode was analyzed. It is used to indicate the degree of aerosol absorption by solar radiation in the ultraviolet band, and it is more sensitive to ultraviolet-absorbing aerosols [32]. The UVAI of absorbing aerosols, such as smoke and mineral dust, is positive, and a small or even negative value represents nonabsorbent aerosols (e.g., sulfates, sea salt, etc.) and clouds [38]. As shown in Figure 6c, the daily mean values of UVAI were low in Siberia before 22 June at ~0.1, indicating the weak absorption of particles in Siberia. With the intensification of the BB and the accumulation of carbonaceous aerosols, the UVAI value in the burning area and its vicinity increased significantly after 22 June, with the highest value reaching 9.5 on 23 June (Figure 8). The daily mean UVAI in Siberia also reached a peak on 24 June of more than six times the daily mean values before 22 June. For the UVAI in Northeast China, the daily mean UVAI was negative at ~−0.1 in Northeast China before the smoky plume’s arrival. The UVAI value in the northern and northeastern regions of Heilongjiang Province rose rapidly from 0.5 to over 2.0 on 25 June, with a maximum of 4.3. After that, the smoke was transported all the way to the south to the Jilin and northern Liaoning provinces. The daily mean UVAI in Northeast China reached a peak of 1.3 on 26 June and subsequently dropped sharply. The drop can be attributed to the retrieval failure of UVAI in the plume-affected area and the aging of the plume. 

**Figure 9:**
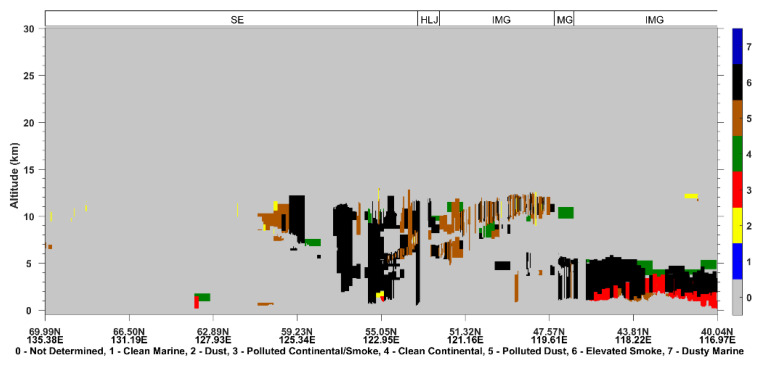
26 June 2017: CALIOP-derived vertical profile of the vertical feature mask of aerosols over the ground track (dashed blue line) shown in Figure 1. CALIOP successively overpassed Siberia (SE), Heilongjiang Province (HLJ), Inner Mongolia Autonomous Region (IMG), Mongolia (MG), and IMG.

In the early morning of 26 June, CALIPSO transited East Siberia, the northern Heilongjiang Province, and eastern Inner Mongolia Autonomous Region (the track position is shown in Figure 1). It can be seen from Figure 9 that the East Siberia area was dominated by smoke aerosols, and the maximum transmission height reached 13 km. From the ground to high altitudes in Heilongjiang Province, there were smoke aerosols and polluted dust (dust mixed with biomass burning smoke [39]) concentrated at 1–11 km. 

**Figure 10:**
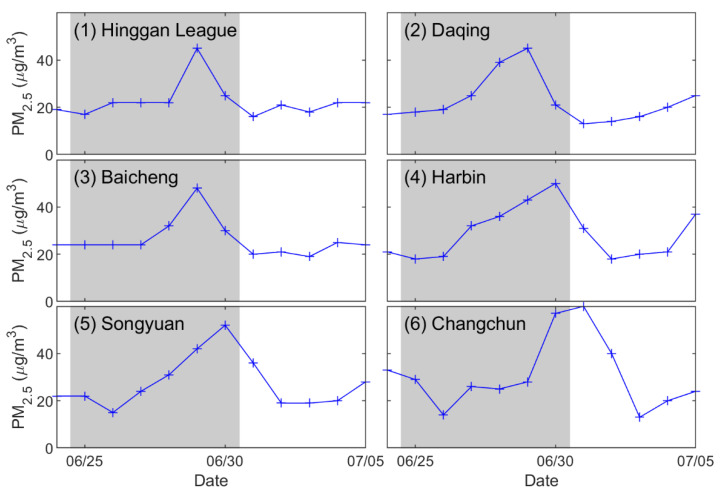
Daily average PM_2.5_ concentration (unit: µg·m^−3^) at the six stations in Northeast China. The biomass burning episode is represented by the grey box.

The altitude of the smoke aerosols in Inner Mongolia transported from Siberia was already close to the ground, and it was more likely to affect the air quality near the ground. This was evidenced by the variation in PM_2.5_ concentrations in Northeast China. The distribution of daily PM_2.5_ concentrations in the six selected cities in Northeast China is shown in Figure 10. It can be found that, after the plume entered Northeast China, the PM_2.5_ concentration increased from 20 µg·m^−3^ to 40 µg·m^−3^ or even higher on 29 or 30 June, when the smoke swept slowly over Northeast China. Generally, it more than double at the six sites.

## 4. Discussions

In recent years, air pollution in Northeast China has attracted more and more attention. In terms of air quality, Northeast China is regarded as the fifth most polluted area in China [40]. Previous studies of the atmospheric environment in Northeast China mostly focus on surface air quality monitoring, assessments of local emissions, and evaluations of regional transport from the North China Plain [41,42,43]. However, little is known about the long-range transport of Siberian smoky plumes on air quality in Northeast China, as they were recognized to significantly affect the atmospheric environment on both regional and hemispheric scales [4]. This study explores the influence of the Siberian plume on air quality in Northeast China in June 2017 based on multiple satellite products and surface PM_2.5_ observations, and the results show that the fires in Siberia emitted large amounts of CO and aerosols into the atmosphere and the smokey plume caused CO concentration, AOD, and UVAI to increase significantly in the plume-affected area in Northeast China. The PM_2.5_ was also doubled near surface in Northeast China after being influenced by the plume. 

A similar episode occurred In July 2014. In July 2014, severe wildfires occurred in the forested regions of East Siberia, and the Siberian plume also caused significant air pollution in Northeast China. The AOD reached 3.0 in most areas in the northern part of Northeast China, and the peak concentration of CO and PM_2.5_ exceeded 5.0×10^18^ molec·cm^2^ and 100 µg·m^−3^ [22], respectively, which are two-three times the values in this study. The air pollution resulted from intense fire activity, approximately 12600 forest fires burned over 1.8 million hectares by 16 July 2014 [21], and consistent transport via northwesterly winds lasted five days [21,22]. In addition, the combustion area in Siberian in July 2014 was more concentrated than that in this study. It indicates the impacts of Siberian plumes on a downwind can vary a lot with emission intensity and downwind meteorology. 

In these two episodes, the northerly wind played an important role in the transport of the Siberian plumes to Northeast China. In both cases, high-pressure ridges near Lake Baikal resulted in the northerly airflow in the north of Northeast China. The northerly flow carried the smokey plume southward. In addition, the northerly airflow in front of the high-pressure ridge is usually accompanied by a downdraft, which is favorable for the subsidence of the pollutants and, thus, influences the air quality near the surface. The forest fires in Siberia are most active in the summertime [44]. When there are BBs in Siberia, more attention should be paid to the meteorological conditions. The suitable downwind meteorology and intense burning can significantly affect the atmospheric environment in Northeast China. 

This study demonstrates that the Siberian smoky plumes can cause regional air pollution in Northeast China under suitable meteorological conditions. The impact of Siberian BB on the atmospheric environment in Northeast China was supported by the enhancement of CO, AOD, UVAI, and PM_2.5_ values during the fire activities in this study. However, owing to the influence of local emissions in Northeast China, a precise evaluation of BB emissions and their impacts on air quality cannot be achieved. Therefore, simulations using chemical transport models are essential to evaluate the impacts of Siberian BBs on the environment. Moreover, global climate change is expected to increase fire activity in Siberia [10]. It is reasonable to expect an increased risk of air quality degradation in Northeast China caused by the long-range transport of Siberian plumes. It is necessary to assess the contribution of long-range transport to air quality in Northeast China. 

## 5. Conclusions

A case study of the long-range transport of a BB plume from Siberia to Northeast China in June 2017 was presented in this study by using multisatellite products and ground-based PM_2.5_ measurements. With the dynamic uplifting, coupled with pyroconvection lifting, the Siberian plume was transported to a high altitude. Moreover, the plume moved eastward and southward, successively, under the high-pressure ridge and the Okhotsk Sea cold vortex systems, affecting the air quality in Northeast China. 

BB in Siberian emitted large amounts of CO and aerosols into the atmosphere. At the early stage of the BB, the daily mean values of the CO column concentration, AOD, and UVAI were 1.6 × 10^18^ molec·cm^2^, 0.2, and 0.1, respectively, in Siberia, and their maximum increased to 1.9 × 10^18^ molec·cm^2^, 0.5, and 0.6 after the BB enhanced. Moreover, their instantaneous maximums reached 7.4 × 10^18^ molec·cm^2^, 4.2, and 9.5 in Siberia.

Before the arrival of the Siberian BB plume, the daily mean values of the CO column concentration, AOD, and UVAI were 1.9 × 10^18^ molec·cm^2^, 0.4, and −0.1, respectively, in Northeast China, and their maximum increased by 6 × 10^17^ molec·cm^2^, 0.5, and 1.4 after being affected. The smoky plume even affected the air quality of the near surface of Northeast China, in which the PM_2.5_ concentrations were doubled or were even higher. 

## Data Availability

The datasets analyzed during this study can be obtained from the corresponding author.
